# Fabricating Microstructures on Freeform Surfaces via Flexible Hydrogel Micromolds

**DOI:** 10.1002/smll.202411751

**Published:** 2025-02-12

**Authors:** Pang Zhu, Zahra Hosneolfat, Niloofar Nekoonam, Sagar Bhagwat, Dorothea Helmer, Bastian E. Rapp

**Affiliations:** ^1^ Laboratory of Process Technology NeptunLab Department of Microsystems Engineering (IMTEK) Albert Ludwig University of Freiburg 79110 Freiburg Germany; ^2^ Freiburg Materials Research Center (FMF) Albert Ludwig University of Freiburg 79104 Freiburg Germany; ^3^ Freiburg Center of Interactive Materials and Bioinspired Technologies (FIT) Albert Ludwig University of Freiburg 79110 Freiburg Germany; ^4^ Glassomer GmbH 79110 Freiburg Germany

**Keywords:** freeform surfaces, microstructured hydrogels, photo‐responsive hydrogels, soft lithography

## Abstract

Fabricating microstructures on curved surfaces is of great importance in several engineering fields, and it is an ongoing challenge to develop technologies that do not require complicated devices and cumbersome procedures. A novel approach is presented for fabricating microstructures with different topographies on curved surfaces using flexible microstructured hydrogels as molds. The hydrogel film is first microstructured via spatially controlled ultraviolet illumination utilizing its photo‐responsive property. The microstructured hydrogel film is then transferred to a desired surface. Due to its low modulus, the hydrogel film can be adapted to the curved surfaces including semi‐cylindrical and semi‐spherical surfaces as well as freeform surfaces with both convex and concave features. The final freeform surfaces with microstructures are obtained by polydimethylsiloxane (PDMS) casting. The resulting microstructures show high uniformity and high surface smoothness with an average roughness (Ra) ≈2.7 nm and a root mean square roughness (Rq) ≈4.1 nm. The ability to fabricate microstructures on large‐area curved surfaces of ≈4.5 cm by 6.5 cm is demonstrated. Finally, it is shown that the fabricated microstructures allow good imaging performance. This method utilizes the hydrogel's photo‐responsive ability to construct flexible molds and flexibility to adapt to freeform surfaces, thereby allowing significantly simplified fabrication processes.

## Graphical abstract

Microstructures on flat hydrogel are created by selective illumination. These microstructured gels can adapt to various curved surfaces owing to their softness and flexibility. PDMS replication creates the final array of microstructures. This strategy alleviates the need for sophisticated equipment and complex procedures to fabricate microstructures on various curved surfaces.

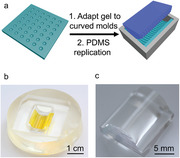

## 1 Introduction

Fabricating microstructures on curved surfaces is highly important in engineering fields, e.g., electronics^[^ ^1^ ^]^ and microoptics.^[^ ^2^ ^]^ However, creating microstructures on curved surfaces is a challenging process that requires expensive instrumentation or cumbersome workflows. Thus, the research for developing simpler methodologies is still ongoing. Various technologies have been suggested to address this challenge. These can generally be divided into two main categories: microstructures are a) directly fabricated on top of curved surfaces, or b) created on flat surfaces and consecutively molded onto a curved surface. Technologies according to a) include ultraprecision diamond turning to directly cut microstructures on curved surfaces.^[^ ^3^ ^]^ For example, Huang et al. fabricated a radial Fresnel lens on a roller mold within 8 h using a four‐axis based tool/workpiece motion, achieving a high‐quality turning surface with an average roughness (Ra) ≈8.25 nm.^[^ ^3a^ ^]^ Lithography, although established for flat surfaces, has also been adapted to curved surfaces.^[^ ^4^ ^]^ As examples, Park et al. and Kim et al. managed to fabricate microstructures on curved surfaces by wrapping a flexible polydimethylsiloxane (PDMS) mask onto a curved surface coated with photo resin. After rotational exposure and development, microstructures on curved surfaces were obtained.^[^ ^4a,b^ ^]^ Li et al. first fabricated a three‐dimensional (3D) microlens array via ultraprecision diamond turning, which was then used as an optical projection system to expose a curved surface achieving microstructure fabrication on curved surfaces.^[^ ^4c^ ^]^ Laser direct writing is another emerging technology for this purpose. Bian et al. fabricated a negative microlens array on a curved glass substrate via a femtosecond laser‐enhanced chemical etching process.^[^ ^5^ ^]^ Jin et al. utilized a femtosecond laser for spatially selective 3D exposure of the photo resin, resulting in negative microstructures on a spherical surface after development.^[^ ^6^ ^]^ These methods share some common drawbacks, i.e., dependence on expensive equipment, laborious procedures, as well as reliance on corrosive solvents. Technologies according to methodology b) include, e.g., wet etching^[^ ^7^ ^]^ or jet printing^[^ ^8^ ^]^ for first creating a flat structured surface. Consecutively, the flat surface is transferred to the curved surface via thermal embossing or pressure‐assisted forming. For example, Deng et al. first fabricated a flat poly(methyl methacrylate) (PMMA) film with microstructures via thermal embossing based on a silica glass master. The flat PMMA was then transferred to a spherical shape via a second thermal embossing by placing the PMMA film on a hot glass bead.^[^ ^7b^ ^]^ While able to produce details on curved surfaces, this technology requires the fabrication of masters by laser etching and two thermal embossing operations, which makes it laborious. Li et al. prepared a flat PDMS film with microstructures using a master fabricated via jet printing. The film was then attached to a microfluidic chamber with negative pressure to deform the film into a concave structure. The final spherical surface with microstructures was then obtained by soft lithography from the deformed film.^[^ ^8a^ ^]^ Next to the limitation in shape by using ink jetting, the control of the final surface shape by negative pressure is not easily adjustable to freeform surfaces.

Hydrogels are frequently used as molds for fabricating microstructures in applications such as microfluidics.^[^ ^9^ ^]^ As examples, Hirama et al.^[^ ^9a^ ^]^ and Sugiura et al.^[^ ^9b^ ^]^ utilized agarose gels as molds to fabricate microchannels. In their approach, agarose gel fibers were arranged manually in a container, and after casting and curing PDMS, the agarose fibers—transformed from solid to liquid under high temperature—were washed out using hot water, resulting in microchannels in the PDMS. Dang et al.^[^ ^9c^ ^]^ fabricated PDMS fluidic devices using hydrogel micromolds prepared via mask‐assisted photopolymerization. On the one hand, they demonstrated that hydrogels can be adapted to various shapes in both two‐dimensional (2D) and 3D spaces owing to hydrogel's softness and flexibility. However, these methods afford relatively low precision, limited to the millimeter scale. On the other hand, hydrogels have been reported as reliable carriers for high‐resolution microstructures. For example, Xiong et al. printed microstructured hydrogels with a resolution of 1 µm via mask‐assisted photopolymerization using a digital micromirror device (DMD).^[^ ^10^ ^]^ Tian et al. fabricated hydrogels with high‐aspect‐ratio micropillar structures via soft lithography showing a resolution of ≈10 µm.^[^ ^11^ ^]^ Our group recently synthesized photo‐responsive hydrogels which can work as micromolds by constructing microstructures on the hydrogel surface via spatially controlled UV illumination.^[^ ^12^ ^]^ The fabricated micromolds demonstrate a high resolution to 30 µm, high surface quality, and potential for optical and microfluidic applications. However, these techniques were limited to fabricating microstructures on flat surfaces or simple cylindrical surfaces.

Here, we report a facile method to prepare microstructures on freeform surfaces based on flexible hydrogel micromolds. A photo‐responsive hydrogel film produced via thermal free radical polymerization is illuminated under UV light using a photo mask, and microstructures on the hydrogel surface are obtained directly without further development or etching procedures. The microstructured hydrogel film is then transferred to a freeform surface, owing to low modulus, the hydrogel film can adapt to various curved surfaces. Finally, the microstructured curved surface is preserved via PDMS replication. Microstructures with different shapes like squares, hexagons, and circles are prepared on curved surfaces including semi‐spherical and semi‐cylindrical surfaces and freeform surfaces. The microstructures are characterized and analyzed using white light interferometry (WLI) and scanning electron microscopy (SEM) showing good structure uniformity. The surface quality is checked via atomic force microscopy (AFM) displaying a smoothness of R_a_ ≈ 2.7 ± 1.1 and R_q_ ≈ 4.1 ± 1.1 nm. In addition, we also show the ability of this strategy to fabricate microstructures on large‐area curved surfaces with an area ≈4.5 cm by 6.5 cm. The sharp images got from fabricated microstructures on a spherical surface demonstrate good imaging performance.

## 2 Results and Discussion

### 2.1 Procedures of Fabricating Microstructures on Curved Surfaces

**Figure 1:**
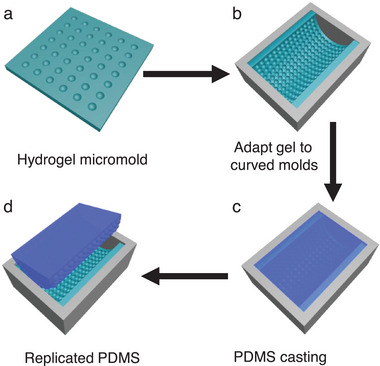
Schematic illustration of fabricating microstructures on curved surfaces. a) The hydrogel micromold with negative microstructures fabricated via a mask‐assisted photolithography process; b) The hydrogel micromold adapts to a curved mold owing to its low modulus thus creating a ready‐to‐use micromold; c) PDMS pre‐polymer is poured onto the hydrogel micromold to replicate the microstructures; d) After curing of PDMS, microstructures on curved surfaces like a semi‐cylindrical surface are obtained by peeling the PMDS from the hydrogel micromold.

To fabricate microstructures on curved surfaces, first, the flat hydrogel micromold was fabricated via a mask‐assisted photolithography process. The process is based on the hydrogel's photo‐responsive property as previously introduced by our group.^[^ ^12^ ^]^ Briefly, the hydrogel shrinks locally under spatially selective UV illumination resulting in negative microstructures on its surface (**Figure** **1**a) without the need for development steps or solvent rinsing. The hydrogel micromold can be used directly after drying the remaining water from the hydrogel surface using a nitrogen gun. Owing to its low modulus, the flat hydrogel micromold can adapt onto various molds such as a semi‐cylindrical mold as shown in Figure 1b, thus creating a ready‐to‐use micromold. PDMS pre‐polymer is then poured onto the adapted hydrogel micromold (Figure 1c). After curing, microstructures on curved surfaces are obtained after peeling the PDMS from the mold (Figure 1d).

### 2.2 Fabricating Microstructures on Regular Curved Surfaces

**Figure 2:**
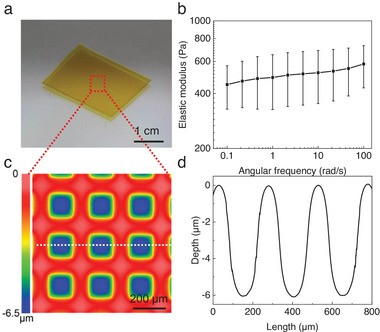
Flat hydrogel micromolds are created by shrinkage induced by selective illumination. a) View of the flat hydrogel illuminated via a photo mask with microsquare patterns (diameter 150 µm, gap 100 µm, Figure S4a, Supporting Information); b) Dynamic rheology of the hydrogel showing elastic modulus (*G′*) as functions of the angular frequency with a value of ≈420 Pa at 0.1 rad s^−1^; c) The 2D topography of microstructures on the flat hydrogel micromold measured by WLI showing microstructures have high uniformity with d) a height of (6.0 ± 0.4 µm) and diameter of (207.5 ± 7.8 µm). Data in (b) is presented as mean values ± standard deviation (SD). Error bars represent the SD from three samples.

Using a photo mask with microsquare patterns (diameter 150 µm, gap 100 µm, Figure S4a, Supporting Information), a flat hydrogel micromold (**Figure** **2**a) was fabricated. Due to the gel's softness, standard tensile tests cannot be executed, as the gel is easily damaged by the high compression force required to fix the samples. Therefore, to determine the elastic modulus of the hydrogel, and frequency sweep tests were conducted finding an elastic modulus of ≈420 Pa at 0.1 rad s^−1^ (Figure 2b). The topography of microstructure of the flat hydrogel micromold was characterized via white light interferometry (WLI, Figure 2c). The obtained microstructures demonstrate high uniformity with a height of (6.0 ± 0.4 µm) and a diameter of (207.5 ± 7.8 µm) (Figure 2d). The topography of the microstructures from another two individual flat hydrogel micromolds was characterized (Figure S1, Supporting Information) finding high consistency among different samples despite of a small height deviation of ≈0.4 µm. In addition, it is worth noting that this kind of method utilizing local swelling or deswelling of the soft substrate can only fabricate microstructures with low aspect ratios from ≈0.03 to 0.25.^[^ ^13^ ^]^

**Figure 3:**
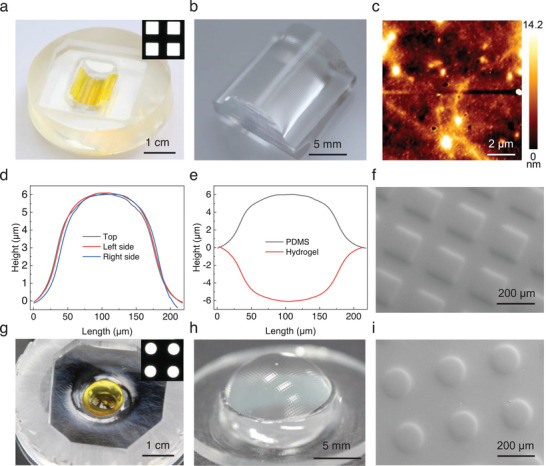
Fabricating microstructure on curved surfaces by flexible hydrogel adaption. a) View of the hydrogel adapted to a semi‐cylindrical mold fabricated via a mask with microsquare patterns (inset, also see Figure S4a, Supporting Information); b) View of the replicated semi‐cylindrical PDMS with microstructures; c) The PDMS replica exhibits high surface quality with a R_a_ ≈2.7 ± 1.1 and a R_q_ ≈4.1 ± 1.1 nm as determined via AFM characterization; d) Profiles of microstructures characterized via WLI on different spots of the PDMS replica shown in (b); e) The comparison between profiles of microstructures on replicated PDMS and the flat hydrogel micromold shown in Figure 1a demonstrating high consistency; f) SEM image of the final PDMS replica; g) View of the hydrogel adapted to a semi‐spherical mold fabricated via a mask with microcircle patterns (inset, also see Figure S4b, Supporting Information); h) The replicated semi‐spherical PDMS with the replicated microstructures; i) SEM image of the final PDMS replica.

To achieve complex surface shapes, the flat hydrogel micromold was placed onto a semi‐cylindrical mold with a diameter of 12 mm (**Figure** **3**a). After instant conformal deformation to the surface curvature, PDMS pre‐polymer was poured into the mold and cured to obtain the final semi‐cylindrical PDMS replica with the desired microstructure (Figure 3b). The PDMS replica shows high surface quality with R_a_ ≈2.7 ± 1.1 and a R_q_ ≈4.1 ± 1.1 nm as determined via AFM characterization (Figure 3c). The topographies of the microstructures at various locations on the PDMS replica were characterized using WLI. As shown in Figure 3d and Figure S2 (Supporting Information), the microstructures exhibited very similar profiles with a height of (6.0 ± 0.4 µm) and diameter of (205.6 ± 9.2 µm), indicating high uniformity. Figure S3 (Supporting Information) shows profiles of microstructures of another two individual PDMS replicas, demonstrating high and precise reproducibility of the method and uniform structures at different locations despite of a small height variation of ≈0.4 µm among the three samples. In addition, a comparison between the profiles of the microstructures on the PDMS replica and those on the flat hydrogel micromold shows that the profiles match, demonstrating that the microstructures were transferred onto the curved PDMS surface with excellent fidelity (Figure 3e). The topography of PDMS replica was further characterized using SEM (Figure 3f), and demonstrates high surface quality. Given the widespread use of PDMS molds in soft lithography, our strategy can be applied to fabricate microstructured surfaces using various materials, such as metals,^[^ ^14^ ^]^ and other curable resins^[^ ^15^ ^]^ by utilizing the PDMS replica as the second mold. This workflow is not limited to cylindrical surfaces. To test the versatility of the method, the fabrication of microstructures of various shapes on a wide range of curved surfaces was tested. As shown in Figure 3g, a flat hydrogel film illuminated under a photo mask with microcircle patterns (diameter 150 µm, gap 150 µm, Figure S4b, Supporting Information) was placed into a semi‐spherical mold (diameter 14 mm). After PDMS casting, microstructures on a semi‐spherical surface were obtained (Figure 3h) with high uniformity (diameter 156 ± 3 µm) and high surface quality with only tiny defects as determined by SEM characterization (Figure 3i).

### 2.3 Fabricating Microstructures on Irregular Curved Surfaces and Large Areas

**Figure 4:**
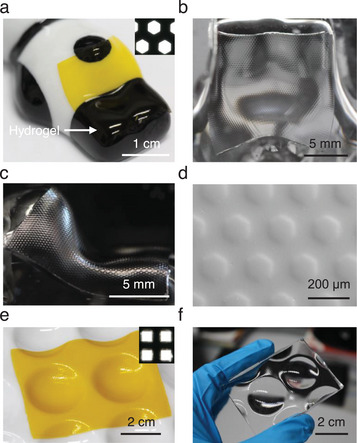
Microstructures fabrication on freeform and large‐area curved surfaces. a) View of the hydrogel micromold fabricated via a mask with microhexagon patterns (inset, also see Figure S4c, Supporting Information) (the hydrogel extends from the yellow area on the white background to the black area as indicated by the arrow) on a ceramic mold with irregular concave and convex features; b,c) View of the final PDMS replica from the top and side view; d) SEM image of the PDMS replica; e) View of the hydrogel micromold (yellow area) fabricated via a mask with microhexagon patterns (inset, also see Figure S4d, Supporting Information) on a large‐area ceramic reaction plate (white color), which functions as a mold; f) View of the final PMDS replica with a size of ≈4.5 × 6.5 cm^2^, demonstrating that the method applies also to large areas.

As stated, fabricating microstructures on irregular curved surfaces is still a significant challenge. To demonstrate the versatility of our method for particularly this purpose, we fabricated microstructures on a freeform surface containing irregular concave and convex topographies using ceramic artwork as the mold. Based on the previously described fabrication procedure, first, the hydrogel micromold illuminated under a photo mask with microhexagon patterns (diameter 120 µm, gap 100 µm, Figure S4c, Supporting Information) was placed onto the ceramic mold. Owing to its inherent softness and flexibility, the hydrogel adapted onto the irregular surface of the ceramic mold (**Figure** **4**a). After PDMS replication, the microstructures were successfully transferred onto the irregular surface (Figure 4b,c), demonstrating high uniformity and surface quality, as confirmed by SEM characterization (Figure 4d). Despite of its adaptability, the hydrogel molds cannot adapt to surfaces with a high curvature. This approach is also suitable for fabricating microstructures on large‐scale curved surfaces. As shown in Figure 4e, the hydrogel micromold illuminated under a laser‐cut photo mask with microsquare patterns (length 530 µm, gap 470 µm, Figure S4d, Supporting Information) was placed onto a ceramic reaction plate. After replicating, a large‐area microstructured PDMS replica (≈4.5 × 6.5 cm^2^) was obtained (Figure 4f).

### 2.4 Imaging Performance

**Figure 5:**
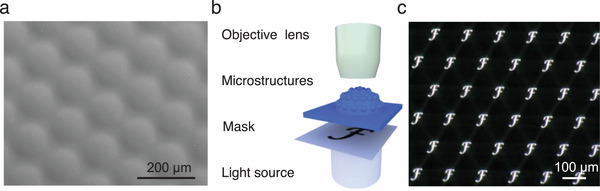
Image performance of exemplary microstructures produced by the hydrogel adaption method on a semi‐spherical surface. a) SEM image of the semi‐spherical PDMS replica; b) Schematic illustration of the setup for imaging performance test; c) Uniform images projected by microstructures on the semi‐spherical surface.

To exemplify the practical applications of our method, we characterized the imaging performance of the microstructures on a semi‐spherical surface. Microhexagon structures were fabricated via a hydrogel micromold illuminated under a photo mask with microhexagon patterns (diameter 80 µm, gap 60 µm, Figure S4e, Supporting Information). The PDMS surface shows high surface quality as seen in SEM characterization (**Figure** **5**a). The setup to check the imaging performance consists of an objective lens and a charge‐coupled device (CCD) camera (Figure 5b). A black film with a transparent letter “F” was placed between the microstructured PDMS replica and a light source. The entire system mimics natural compound eyes, where the microstructures on the curved surface function as ommatidia, capturing environmental light signals, while the camera acts as the visual nervous system, receiving and processing these signals to produce final images.^[^ ^16^ ^]^ As can be seen from Figure 5c, the microstructures on the surface can project a sharp image resulting in multiple replicas of the letter “F”. This demonstrates the excellent optical properties of the replicated components and thus underlines the potential application of this method in imaging applications.

## 3 Conclusion

In this paper, we developed a facile method to fabricate high‐resolution microstructures on freeform surfaces using flexible hydrogel micromolds. This approach utilizes the photoresponsive property of the custom‐synthesized hydrogel to create micromolds directly without the need of any further development or etching. The low modulus of the fabricated hydrogel micromolds allows them to adapt to both regular and irregular freeform surfaces. Using this process, we successfully fabricated microstructures on curved surfaces with high uniformity including cylindrical, spherical, and irregular geometries. We also demonstrate the creation of large‐area microstructured curved surfaces of several centimeters in size. The fabricated microstructures demonstrated good imaging performance. This method offers a promising solution for microstructure fabrication on curved surfaces without the need for sophisticated equipment and complex procedures.

## 4 Experimental Section

### Materials

Acrylamide (AM, 99%), α‐cyclodextrin (α‐CD, 99%), 4‐phenylazopheonl (98%), methacrylic anhydride (94%), ammonium peroxydisulphate (APS, 98%), N,N′‐methylenebisacrylamide (MBA, 99%), 4‐dimethylaminopyridine (DMAP, 98%), sodium chloride (NaCl, 99%), magnesium sulfate (MgSO_4_, 98%) were purchased from Sigma–Aldrich, Germany. Methanol (99.9%), dimethyl sulfoxide (DMSO, 99.5%), and tetrahydrofuran (THF, 99.5%) were purchased from Carl Roth, Germany; 1*H*,1*H*,2*H*,2*H*‐perfluoroctyldimethylchlorosilane (97%) was purchased from Abcr GmbH, Germany. Polydimethylsiloxane (PDMS, Elastosil 601A, and 601B) was purchased from Wacker, Germany. A transparent silicon rubber sheet (thickness 0.3 mm) was purchased from Technikplaza, Germany. Photo masks were partially purchased from KOENEN GmbH and partially cut by a laser cutting machine.

### Preparation of AM/AZO‐CD Photo‐Responsive Hydrogels

4‐methacryloyloxy azobenzene (AZO) monomer was synthesized according to a protocol previously reported by the group.^[^ ^12^ ^]^ AM/AZO‐CD hydrogels were prepared by dissolving 15 mmol AM, 0.6 mmol AZO monomer, 0.15 mmol MBA, and 0.09 mmol APS in 4 mL DMSO to obtain a clear pre‐gel solution. The solution was injected into a sandwich glass cell composed of two functionalized glass slides. The slides were treated to obtain increased surface hydrophobicity^[^ ^17^ ^]^ and a silicon rubber spacer (thickness 0.3 mm) was used to create a volume between them. The polymerization was carried out in an oven at 45 °C for 24 h. After removing the glass slide, the gel was immersed into α‐CD/H_2_O solution (25 mg mL^−1^) for 24 h to reach a fully swollen state before use.

### Fabrication of Microstructures on Curved Surfaces

To construct microstructures on the flat hydrogel film, the swollen hydrogel film was placed on a glass slide and covered with a cover slide (thickness 0.1 mm). The hydrogel film is vulnerable owing to its low modulus, careful handlining using tweezers equipped with soft plastic tips can help to prevent breakage. A photo mask was placed onto the cover slide and the hydrogel film was illuminated by UV light (wavelength: 320–400 nm, light intensity: 24.1 mW cm^−2^). After 10 min exposure, the remaining water on the hydrogel surface was gently removed using a nitrogen gun to obtain the microstructured hydrogel. Then, the structured hydrogel was placed onto pre‐prepared curved surfaces. Liquid PDMS pre‐polymer was mixed and degassed according to the manufacturer and poured onto the hydrogel micromold and left to cure overnight at room temperature. Subsequently, the cured PDMS was peeled off to obtain curved surfaces with microstructures.

### Characterization

The topography of the microstructured flat hydrogel films was characterized using white light interferometry (WLI, Newview 9000, Zygo, Germany). Microstructure topography of replicated PDMS was characterized using WLI and scanning electron microscope (SEM, Tescan Amber X, Czech Republic). Owing to the limitation of WLI with measuring microstructures on curved surfaces, only a small area could be focused, therefore, the microstructures on the replicated PDMS were scanned individually. The surface smoothness of replicated PDMS was determined via atomic force microscopy (AFM) (JPK Nanowizard 4, Bruker, Germany); the roughness tests were conducted on semi‐cylindrical samples shown in Figure 3b, and a standard “surface flattening” (also referred to as “background subtraction” or “polynomial leveling”) procedure within the AFM software was applied, which allows the subtraction of the macroscopic shape (i.e., the cylindrical or spherical curvature) so that only the intrinsic micro‐scale surface roughness remains in the analyzed data. Frequency sweep tests (0.1–100 rad s^−1^) were performed on a stress‐controlled rheometer MCR302 (Anton Paar, Germany). Samples were tested at 20 °C using a parallel plate geometry (diameter: 25 mm) at a fixed strain of 1% which is in the linear viscoelastic regime of samples as determined by amplitude sweep measurements. Imaging performance was characterized using a microscope with a CCD camera (VHX‐6000, Keyence Deutschland Gmbh).

## Conflict of Interest

The authors declare no conflict of interest.

## Supporting information



Supporting Information

## Data Availability

The data that support the findings of this study are available from the corresponding author upon reasonable request.
