# Supplementary material for: Fabricating Microstructures on Freeform Surfaces via Flexible Hydrogel Micromolds
Source: Small. 2025 Feb 12;21(11):2411751. doi: 10.1002/smll.202411751 (PMC11922019; doi:10.1002/smll.202411751)
Supplement: Supplementary file 1 — Supporting Information [file SMLL-21-2411751-s001.docx]

Supporting Information

Fabricating Microstructures on Freeform Surfaces via Flexible Hydrogel Micromolds

Pang Zhu, Zahra Hosneolfat, Niloofar Nekoonam, Sagar Bhagwat, Dorothea Helmer*, Bastian E. Rapp


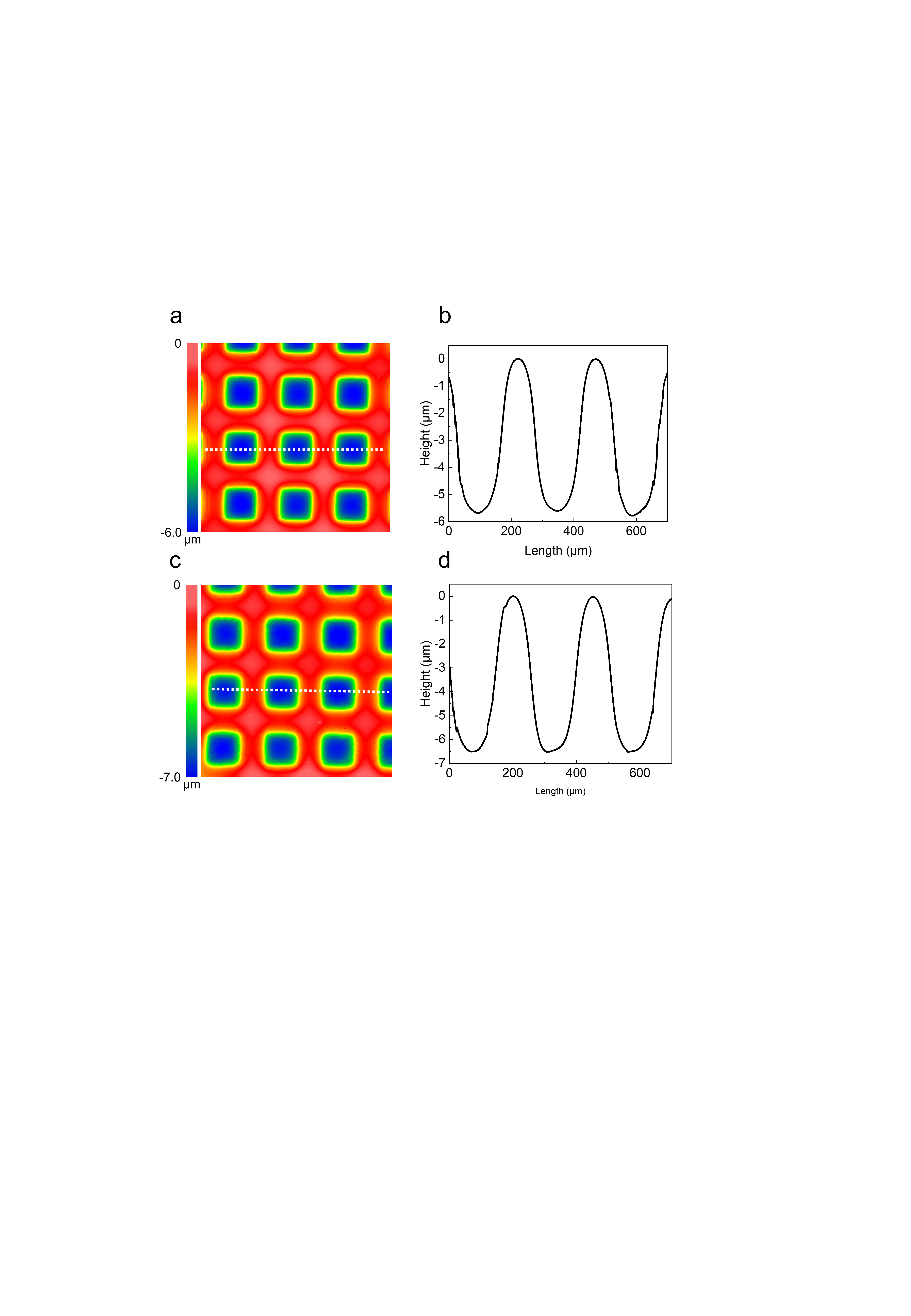


**Figure S1.** The two-dimensional topography of microstructures from two additional individual hydrogel micromolds measured by WLI showing high uniformity compared with data shown Figure 2c & d despite of small height deviation of about 0.4 µm among the three samples.


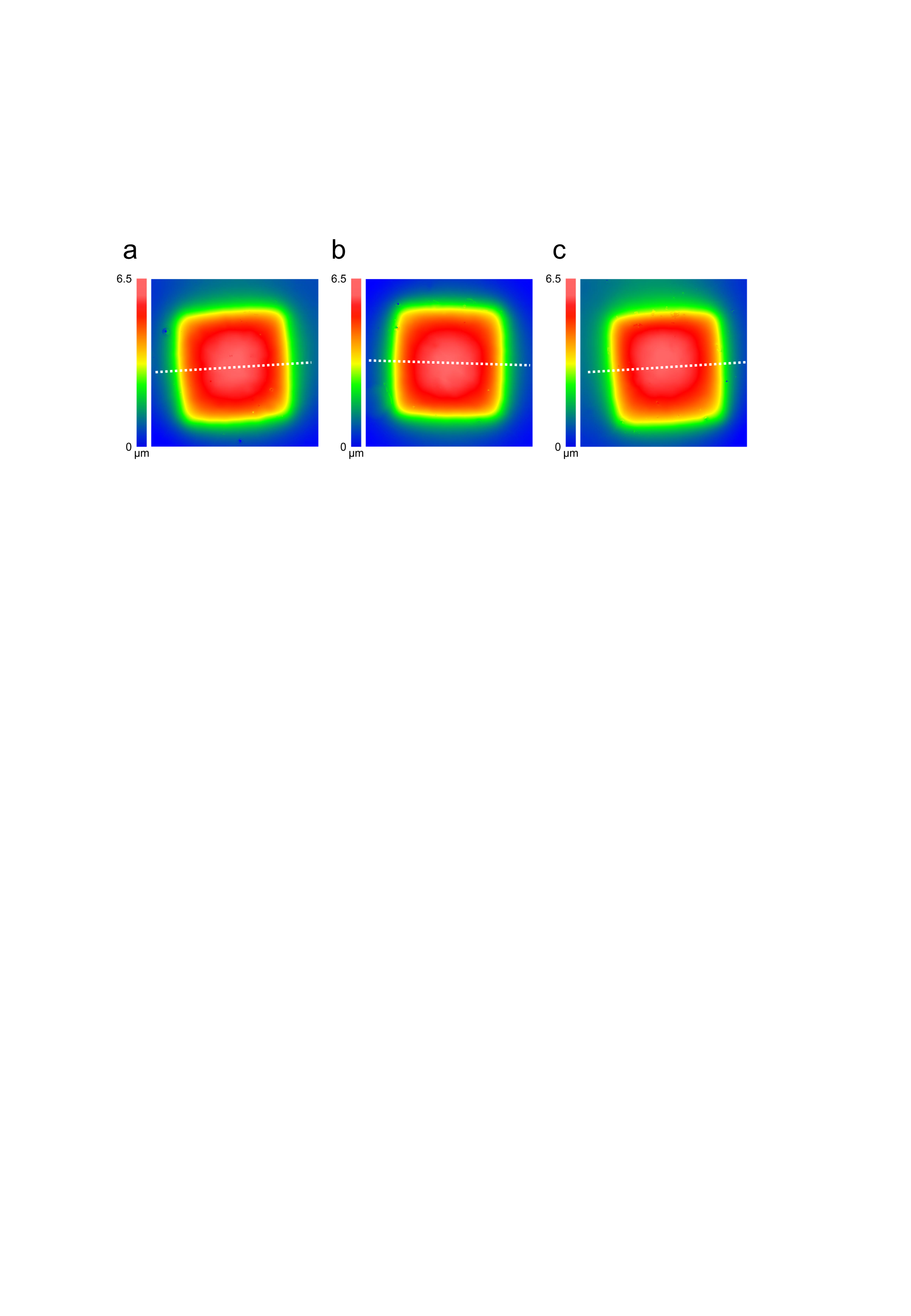


**Figure S2.** Two-dimensional topography of microstructures distributed on different spots ——from (a) left part to (b) top part to (c) right part—— of the semi-cylinder PDMS replica shown in Figure 3b; The white dash line represents where the profile shown in Figure 3d was measured.


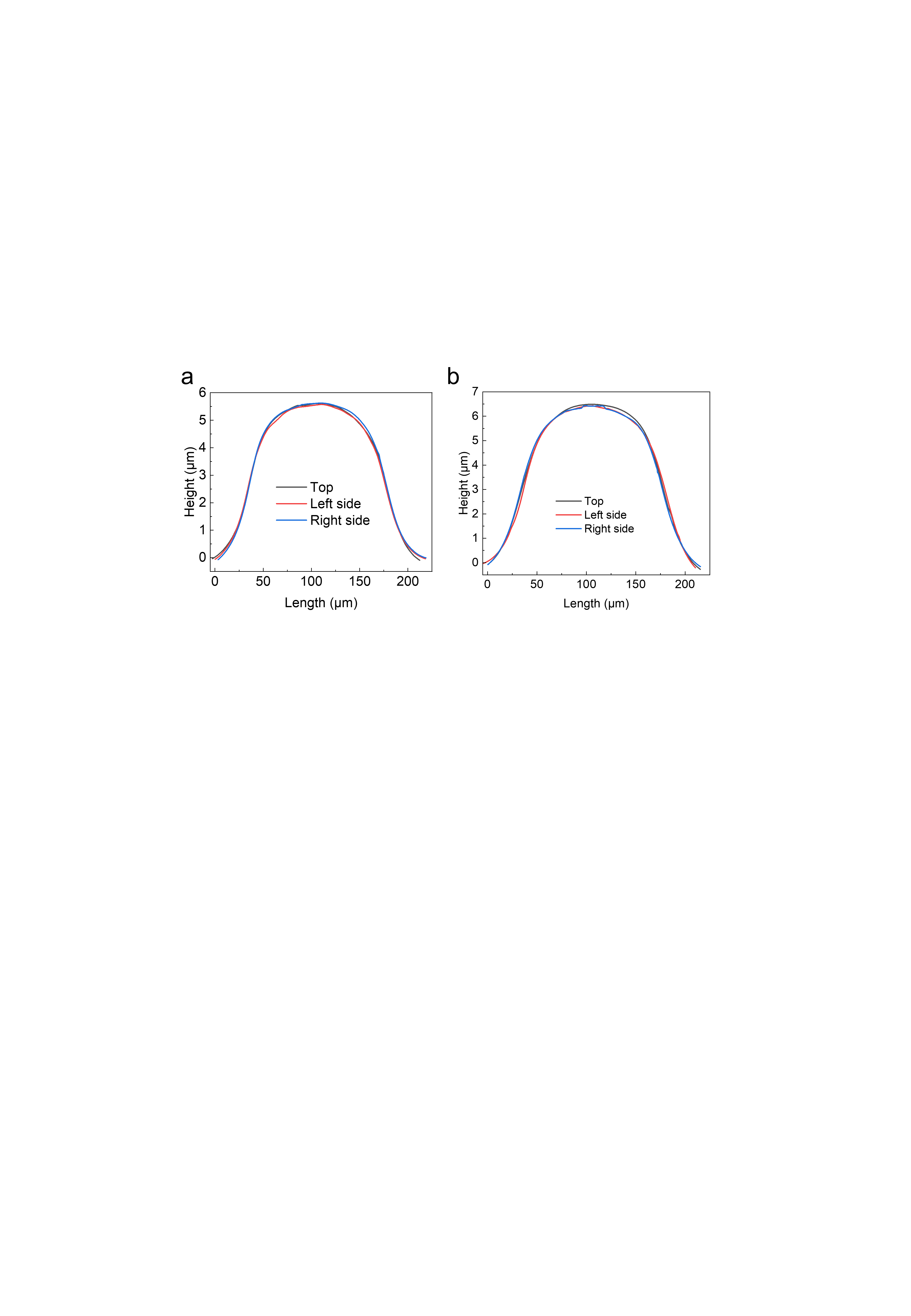


**Figure S3.** Profiles of microstructures characterized via WLI on different spots of the PDMS replica from two additional samples, demonstrating uniform microstructures on different areas of PDMS replicas and consistency among different samples.


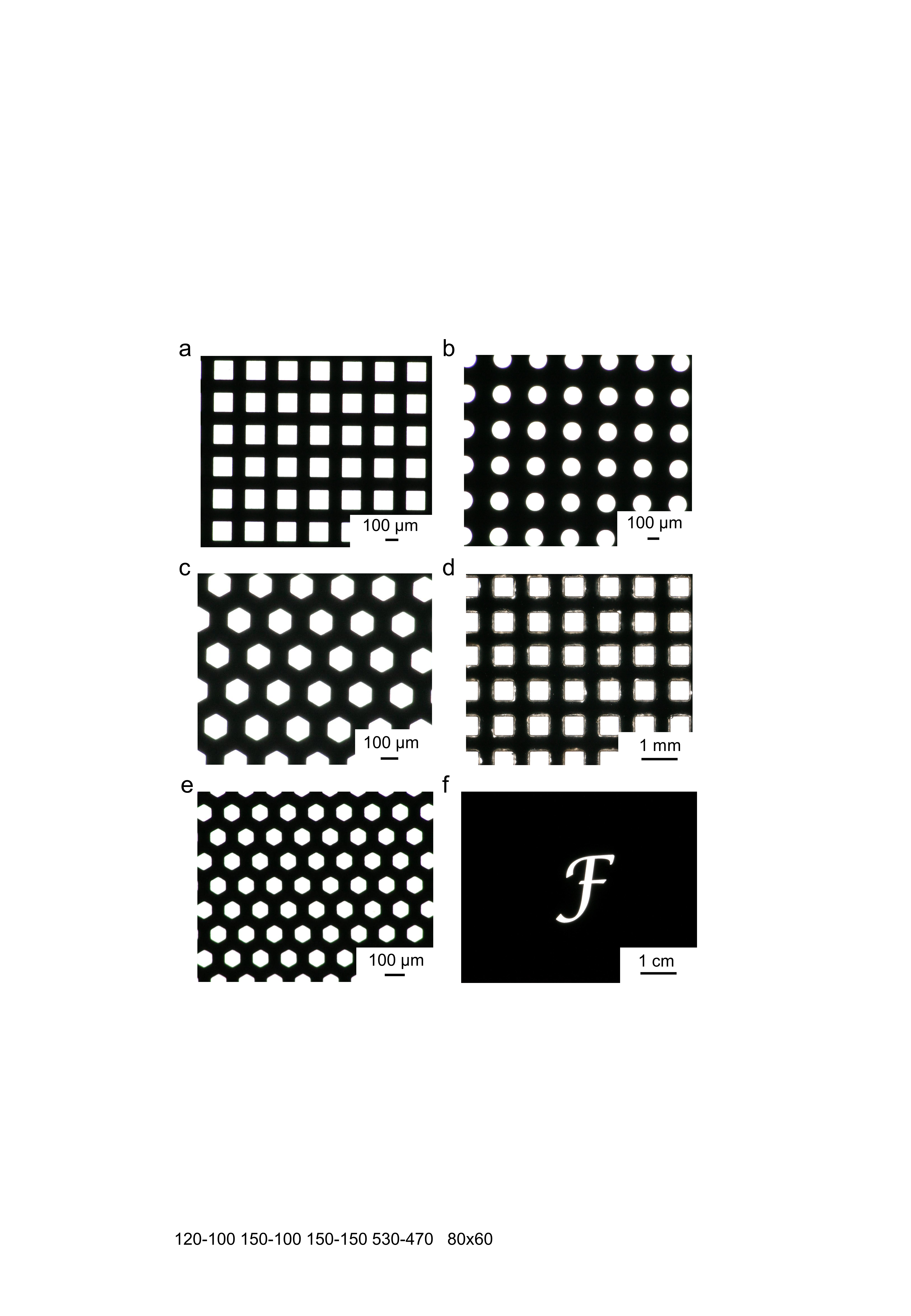


**Figure S4.** Photo masks employed for fabricating microstructures and imaging test. (a) A microsquare array: length 150 μm, gap 100 μm; (b) A microcircle array: circle diameter 150 μm, gap 150 μm; (c) A microhexagon array: hexagon diameter 120 μm, gap 100 μm; (d) A laser-cut mask with a microsquare array: squares 530 μm, gap 470 μm; (e) A microsquare array: square diameter 80 μm, gap 60 μm; (f) A mask with a letter “F”.
